# Synchronous Ipsilateral Wilms’ Tumor and Neuroblastoma in an Infant

**Published:** 2016-01-01

**Authors:** Yogesh Kumar Sarin, Nirali Chirag Thakkar, Shalini Sinha

**Affiliations:** Department of Pediatric Surgery, Maulana Azad Medical College and Lok Nayak Hospital,New Delhi, India

**Keywords:** Synchronous, Wilm's tumor, Neuroblastoma

## Abstract

Wilms’ tumor (WT) and neuroblastoma (NB), the two most common extra-cranial solid malignant tumors, are seldom seen together in the same patient. A 10-month girl presented with a right retroperitoneal mass. A preoperative diagnosis of Wilms’ tumor (WT) was made. She was given preoperative chemotherapy followed by surgery. At surgery a renal mass (WT) and a suprarenal mass (neuroblastoma – NB) were removed. She finally succumbed to metastatic NB in the postoperative period.

## INTRODUCTION

Simultaneous occurrence of WT and NB often leads to confusion in diagnosis, staging, and management. No clear guidelines are available about which malignancy to target first with the appropriate adjuvant therapy. Here we present a rare case of synchronous WT and NB in a girl.

## CASE REPORT

A 10-month old girl presented with history of abdominal distension since 45 days associated with fever, cough and weight loss. There was a history of malignancy in multiple members of the maternal side of the family; however the exact details were not available. On examination a right-sided abdominal mass was found. On ultrasonography a 9 cm x 7 cm heterogeneous predominantly echogenic mass with multiple internal anechoic areas along anterolateral aspect of right kidney, causing its posterior displacement was reported. There was another well-defined 4.8 cm x 4.3 cm heterogeneous, predominantly echogenic mass in right suprarenal region causing inferior displacement of the kidney. Contrast enhanced computed tomography (CECT) showed a similar picture of the two masses (Fig. 1). Chest radiograph showed no lesion. Trucut biopsy from the mass showed tubular and papillary structures lined by tall columnar cells, showing nuclear hyperchromasia with atypia and mitosis, suggestive of epithelial component of WT.

**Figure:**
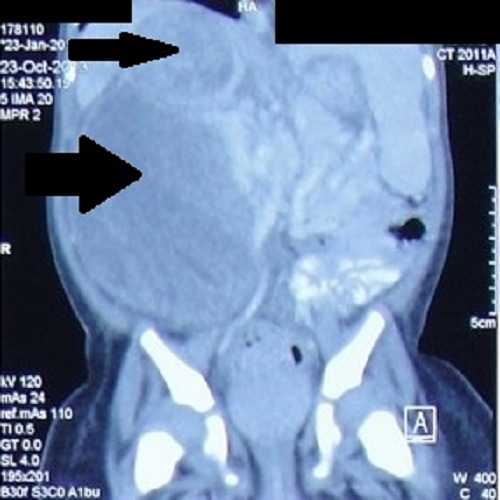
Figure 1:CECT showing two heterogeneous masses in right renal (big arrow) and suprarenal (small arrow) locations respectively.

As per SIOP protocol, a diagnosis of WT (localized disease) was made and the patient was given vincristine and actinomycin-D for four weeks. Repeat CECT did not show significant reduction in tumor size. Surgery was then planned. On exploration, there was a 15 cm x 10 cm x 8 cm mass involving the right kidney, and another 5 cm x 5 cm x 3 cm mass located separately above the right kidney in the region of suprarenal gland (Fig. 2). Enlarged supra-hilar, hilar, infra-hilar and mesocolic lymph nodes were also found. Patient underwent right nephroureterectomy with excision of suprarenal mass and lymph node sampling. There was no tumor spill. In postoperative period patient developed adhesive intestinal obstruction requiring repeat exploration with adhesiolysis on day 5.

**Figure:**
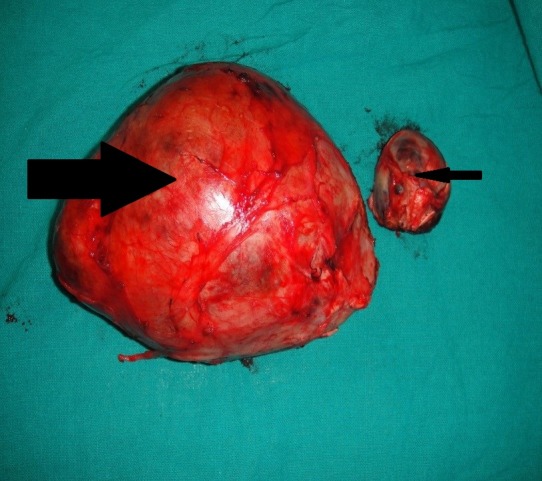
Figure 2:Renal mass (big arrow) and suprarenal mass (small arrow)

The final histopathology report showed WT of the right kidney with predominantly epithelial component (resected margins were free of the tumor) (Fig. 3). The right suprarenal mass showed undifferentiated NB involving the capsule (Fig. 4). The hilar and infra-hilar nodes showed tumor deposits of WT. A thorough work-up for risk factors and metastasis of neuroblastoma was done. It included skeletal survey, meta-iodobenzyl guanidine (MIBG) scan, 24-hour urinary vanillyl mandelic acid levels and bone marrow aspiration biopsy. They were within normal limits. However, N-myc amplification in tumor tissue was positive. Thus, a final diagnosis of WT stage III (intermediate risk) with NB stage I (high risk) was made. The WT was of higher stage and was thus targeted first with radiotherapy (21 Gy radiation over 5 weeks) given to tumor bed with opposite kidney shielding. The patient was then put on vincristine (oncovin), cisplatin, etoposide and cyclophosphamide (OPEC chemotherapy) for NB. During the second cycle of chemotherapy she developed metastatic NB in the left supraclavicular lymph nodes. Repeat CECT scan showed marked retroperitoneal lymphadenopathy and MIBG scan showed suspicious increased uptake in left superior mediastinum. Thereafter, the child had a stormy clinical course. She developed left sided hemorrhagic pleural effusion (cytology showed reactive mesothelial cells in hemorrhagic background) with respiratory distress that was not responding to intercostal drain insertion. In view of unresponsiveness to OPEC, she was begun on rapid COJEC (cisplatin, oncovin, carboplatin, etoposideand cyclophosphamide) chemotherapy. However, her clinical condition continued to deteriorate. She developed decreased oral intake as well as axillary lymphadenopathy followed by abdominal distension and finally succumbed to metastasis.

**Figure:**
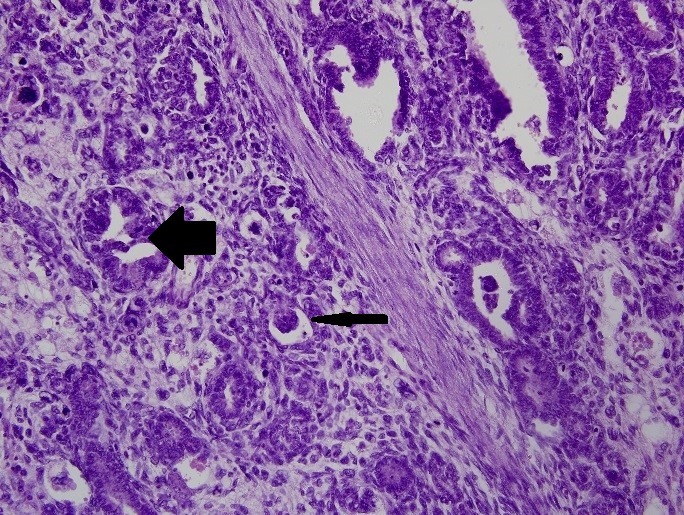
Figure 3:Histopathological examination of renal mass showing abortive tubule formation (big arrow) as well as glomeruli formation (small arrow), suggestive of WT

**Figure:**
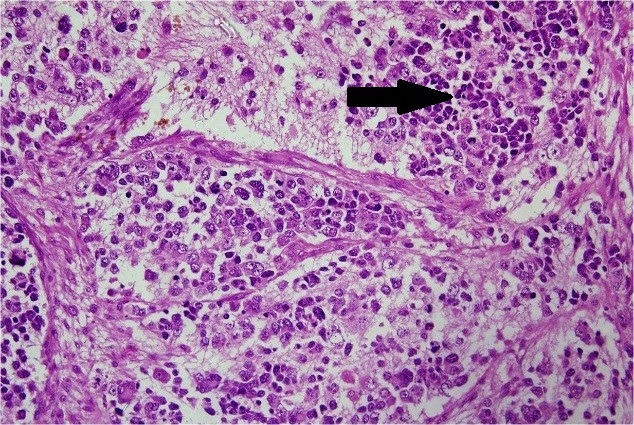
Figure 4:Histopathological examination of suprarenal mass showing round blue cell tumor (arrow). Immunohistochemistry was suggestive of undifferentiated NB

## DISCUSSION

There are few case reports of the simultaneous occurrence of WT and peripheral neuroectodermal tumors (PNET).[1-6] All reported cases of WT associated with NB died. There is also a strong association with vertebral, anorectal, cardiac, tracheoesophageal, renal and limb anomalies (VACTERL association) as well as with Fanconi’s anemia (FA). FA is a genetic (autosomal recessive) disorder, which manifests in childhood with various developmental defects.[7] It is associated with an increased risk of solid malignancies.[8]

There was difficulty in the preoperative diagnosis as two growths were mistaken as one in spite of imaging. Thus biopsy of only the renal mass was taken. This led to a significant delay in the diagnosis of NB and initiation of appropriate chemotherapy. Although the WT was stage III and NB was only stage I, the patient finally succumbed because of NB. This highlights the negative effect of N-myc amplification on the prognosis.

## Footnotes

**Source of Support:** Nil

**Conflict of Interest:** None declared

